# Crystal structure of diethyl 2-acet­oxy-2-[3-(4-nitro­phen­yl)-3-oxo-1-phenyl­prop­yl]malonate

**DOI:** 10.1107/S2056989016001432

**Published:** 2016-01-27

**Authors:** Nóra Veronika May, Gyula Tamás Gál, Zsolt Rapi, Péter Bakó

**Affiliations:** a Institute of Organic Chemistry, Research Centre for Natural Sciences, Hungarian Academy of Sciences, H-1519 Budapest, POB 206, Hungary; b Department of Organic Chemistry and Technology, Budapest University of Technology and Economics, H-1521 Budapest, POB 91, Hungary

**Keywords:** crystal structure, phenyl­propyl malonate, phase-transfer reaction, crown ether catalyst, hydrogen bonding, C—H⋯π ring inter­actions

## Abstract

In the racemic title compound, C_24_H_25_NO_9_, the dihedral angle between the planes of the two benzene-ring systems is 80.16 (6)°, while the side-chain conformation is stabilized by a methyl­ene–carboxyl C—H⋯O hydrogen bond. Weak inter­molecular C—H⋯O hydrogen bonds form inversion dimers [graph set *R*_2_^2^(16)] which are linked into chains extending along *a*. Further C—H⋯O hydrogen bonding extends the structure along *b* through cyclic *R*_2_^2^(10) motifs. Although no π–π aromatic ring inter­actions are present in the structure, C—H⋯π ring inter­actions across *c* generate an overall three-dimensional supra­molecular structure.

## Abstract 2

The title compound diethyl 2-acet­oxy-2-[3-(4-nitro­phen­yl)-3-oxo-1-phenyl­prop­yl]malonate possesses a three-dimensional supra­molecular structure formed through weak C—H⋯O and C—H⋯π hydrogen bonds.

## Chemical context   

The formation of C—C bonds by the Michael addition of the appropriate carboanionic reagents to α,β-unsaturated car­bonyl compounds is one of the most useful methods of remote functionalization in organic synthesis (Mather *et al.*, 2006 ▸; Little *et al.*, 1995 ▸). In particular, a much studied reaction is the conjugate addition of malonates to chalcones. Compounds with the chalcone backbone were reported to possess a wide range of biological activities, such as nematicidal, anti­fungal, anti­allergenic, anti­microbial, anti­cancer, anti­malarial and anti­feedant properties. Malonates are traditionally regarded as important materials for synthesizing the key inter­mediates of numerous active substances, but are rarely found as pharmacophores belonging to the target compounds (Lopez *et al.*, 2001 ▸; Chen *et al.*, 2016 ▸). Therefore, a catalytic version of the Michael addition of dialkyl malonates to chalcones in the presence of different catalysts has been studied extensively in recent years. Many phase-transfer-catalyzed methods that are simple and environmentally friendly have been developed for the Michael reaction (Shioiri, 1997 ▸). This new racemic compound was prepared in a phase-transfer reaction using a sugar-based crown ether as the catalyst (Rapi *et al.*, 2016 ▸).
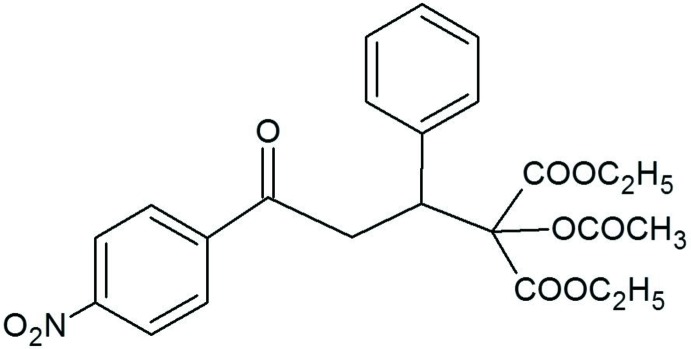


## Structural commentary   

**Figure 1:**
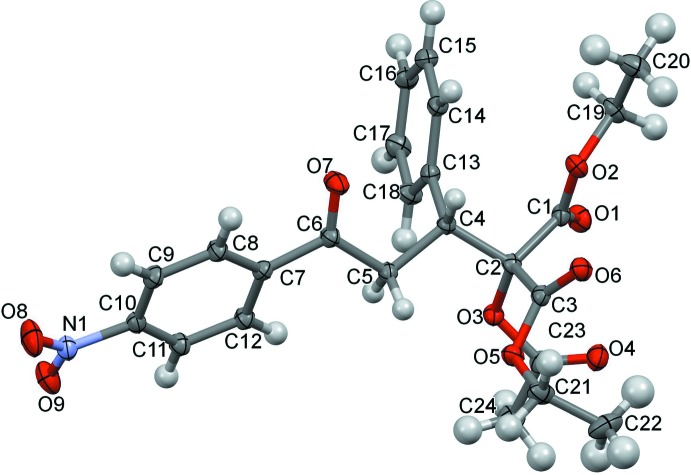
The mol­ecular structure of the title compound, showing the atom labelling. Displacement ellipsoids are drawn at the 50% probability level.

The mol­ecular structure of the racemic title compound is shown in Fig. 1 ▸. In this mol­ecule, the C4 atom is a chiral centre, but no resolution occurred upon crystal preparation, the racemic mixture crystallizing in the centrosymmetric space group *P*2_1_/*n*. The dihedral angle between the planes of the two benzene rings is 80.16 (6)° and the mol­ecular conformation is stabilized by an intra­molecular methyl­ene C5—H⋯O5 hydrogen bond (Table 1 ▸).

## Supra­molecular features   

**Figure 2:**
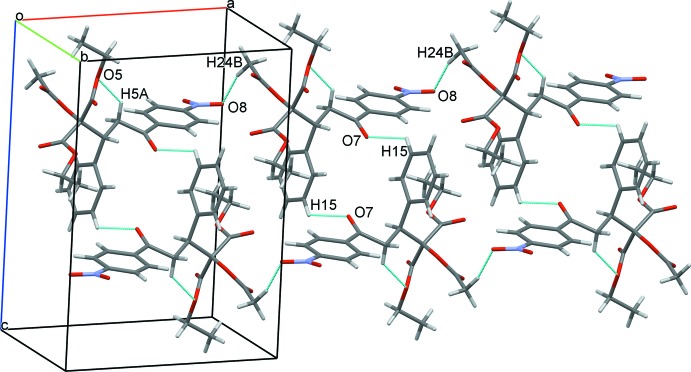
A view of the column structure extending along the *a* axis, showing the C—H⋯O inter­actions as dashed lines.

**Figure 3:**
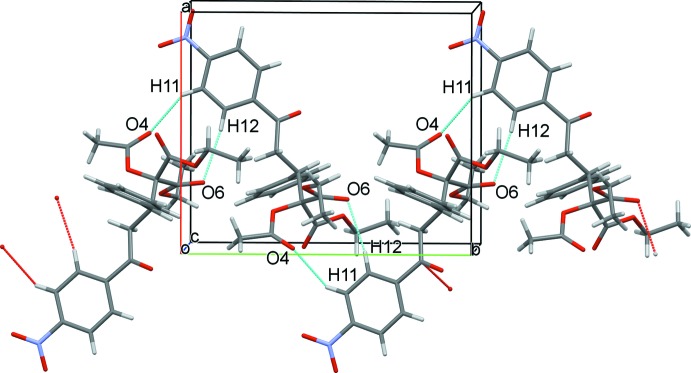
A view of the column expansion along the *b* axis, showing the C—H⋯O inter­actions as dashed lines.

**Figure 4:**
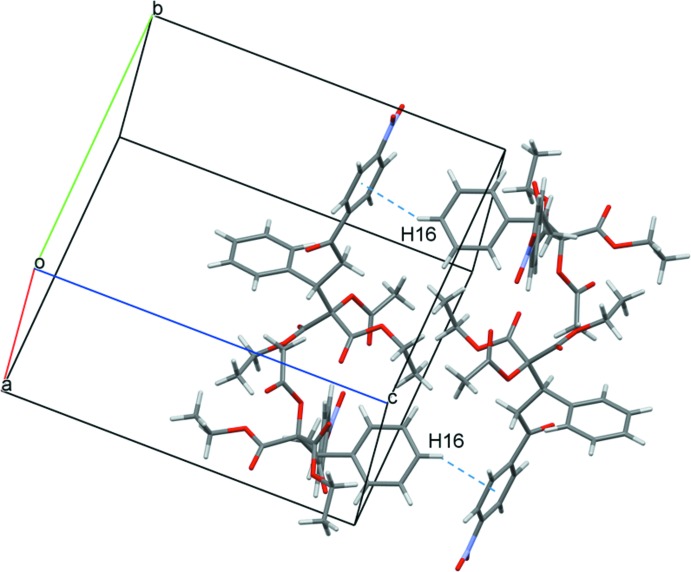
The arrangement of four mol­ecules, showing the C—H⋯*Cg* inter­actions (dashed lines).

Because of the numerous C=O acceptor groups and the lack of primary donor groups in the mol­ecule, the main inter­molecular inter­actions in the crystal are weak C—H⋯O_carbox­yl_ hydrogen bonds (Table 1 ▸), having H⋯O distances equal to or less than 2.6 Å. However, one of the four inter­actions (C24—H⋯O8^iii^; see Table 1 ▸ for hydrogen-bond geometry details and symmetry codes) involves a nitro O-atom acceptor. Inter­molecular C15—H⋯O7^ii^ hydrogen bonds form centrosymmetric cyclic dimers (Fig. 2 ▸) having the graph-set descriptor (Bernstein *et al.*, 1995 ▸) 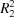
(16). These dimers are linked along the crystallographic *a* direction through C24—H⋯O8^iii^ hydrogen bonds, forming a chain. These chains are further extended in the crystallographic *b* direction through C11—H⋯O4^i^ and C12—H⋯O6^i^ inter­actions, forming a cyclic motif with the graph-set descriptor of 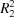
(10) (Fig. 3 ▸). Despite the presence of two aromatic rings in the mol­ecule, there are no significant π–π inter­actions in the crystal lattice. This can be explained by the diverse chain system of the mol­ecule and, therefore, the steric preference of the C—H⋯O hydrogen bonds. However, there is a C16—H16⋯π inter­action across *c* with the C7–C12 nitro­phenyl ring (C⋯*Cg* ^iv^ = 2.81 Å and C—H⋯*Cg* ^iv^ = 149°; *Cg* is the centroid of the C7–C12 ring) (Fig. 4 ▸ and Table 1 ▸), resulting in an overall three-dimensional supra­molecular structure. The relatively high calculated density (1.367 Mg m^−3^) and KPI index (Kitaigorodskii packing coefficient = 69.6%) (Spek, 2009 ▸) show efficient packing of the mol­ecule, resulting in no residual solvent-accessible voids.

## Database survey   

The structures of different derivatives of 1,2-di­phenyl­pentan-1-one, carrying methyl or nitrile substituents on the chiral C atom, have been reported, *viz.* Cambridge Structural Database (CSD; Groom & Allen, 2014 ▸) refcodes RULFIN [(*S*)-4-methyl-4-nitro-1,3-di­phenyl­penta­none; Bakó *et al.*, 1997 ▸], DULJOK (1,3-di­phenyl­butan-1-one; Bąkowicz & Turowska-Tyrk, 2010 ▸) and LAPKEU (4-oxo-2,4-di­phenyl­butano­nitrile; Abdel-Aziz *et al.*, 2012 ▸). RULFIN and DULJOK crystallized in the chiral *P*2_1_2_1_2_1_ and *Pca*2_1_ space groups, respectively, and LAPKEU crystallized as a racemic mixture in the centrosymmetric *P*2_1_/*c* space group. Comparing the dihedral angles between the planes of the two benzene rings, the steric effect of the bulky substituents on atom C2 can be seen. This value is 62.5° for the methyl derivative (DULJOK) and 68.4° for the nitrile (LAPKEU), but significantly higher for the bulky meth­yl–nitro derivative (88.13°; RULFIN) or the title compound (80.2°).

## Synthesis and crystallization   

The title compound was synthesized by the reaction of 4′-nitro­chalcone [(*E*)-3-(4-nitro­phen­yl)-1-phenyl­prop-2-en-1-one] with diethyl 2-acet­oxy­malonate. The reaction was carried out in a solid/liquid two-phase system [Na_2_CO_3_/tetra­hydro­furan (THF)] in the presence of a gluco­pyran­oside-based crown ether catalyst. The compound was isolated by preparative thin-layer chromatography (TLC) (silica gel) in good yield. The structure of the compound was confirmed by ^1^H and ^13^C NMR and mass spectroscopy (m.p. 366–369 K). The details of the synthesis are presented in Rapi *et al.* (2016 ▸). Single crystals of the title compound suitable for X-ray diffraction analysis were obtained by crystallization from ethanol.

## Refinement   

Crystal data, data collection and structure refinement details are summarized in Table 2 ▸. All H atoms were located in difference electron-density maps. However, these atoms were included in the structure refinement at calculated positions, with C—H = 0.95–1.00 Å, and allowed to ride, with *U*_iso_(H) = 1.2*U*_eq_(C).

## Supplementary Material

Crystal structure: contains datablock(s) I, header. DOI: 10.1107/S2056989016001432/zs2354sup1.cif

Structure factors: contains datablock(s) I. DOI: 10.1107/S2056989016001432/zs2354Isup2.hkl

Supporting information file. DOI: 10.1107/S2056989016001432/zs2354Isup3.cml

CCDC reference: 1449223

Additional supporting information:  crystallographic information; 3D view; checkCIF report

## Figures and Tables

**Table 1:** Hydrogen-bond geometry (Å, °) *Cg* is the centroid of the C7–C12 ring.

*D*—H⋯*A*	*D*—H	H⋯*A*	*D*⋯*A*	*D*—H⋯*A*
C5—H5*A*⋯O5	0.99	2.41	3.1403 (15)	130
C11—H11⋯O4^i^	0.95	2.54	3.2588 (16)	133
C12—H12⋯O6^i^	0.95	2.56	3.4879 (15)	165
C15—H15⋯O7^ii^	0.95	2.60	3.2038 (17)	122
C24—H24*B*⋯O8^iii^	0.98	2.47	3.402 (2)	158
C16—H16⋯*Cg* ^iv^	0.95	2.81	3.6550 (14)	149

Symmetry codes: (i) 
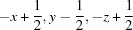
; (ii) 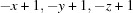; (iii) 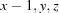; (iv) 
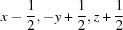
.

**Table 2:** Experimental details

Crystal data	
Chemical formula	C_24_H_25_NO_9_
*M*_r_	471.46
Crystal system, space group	Monoclinic, *P*2_1_/*n*
Temperature (K)	103
*a*, *b*, *c* (Å)	11.0111 (7), 13.1762 (8), 15.8196 (9)
β (°)	93.802 (2)
*V* (Å^3^)	2290.1 (2)
*Z*	4
Radiation type	Mo *K*α
μ (mm^−1^)	0.11
Crystal size (mm)	0.45 × 0.38 × 0.08

Data collection	
Diffractometer	R-AXIS RAPID
Absorption correction	Empirical (*NUMABS*; Higashi, 2002 ▸)
*T*_min_, *T*_max_	0.957, 0.979
No. of measured, independent and observed [*I* > 2σ(*I*)] reflections	67635, 7609, 6054
*R*_int_	0.046
(sin θ/λ)_max_ (Å^−1^)	0.735

Refinement	
*R*[*F* ^2^ > 2σ(*F* ^2^)], *wR*(*F* ^2^), *S*	0.049, 0.128, 1.12
No. of reflections	7609
No. of parameters	310
H-atom treatment	H-atom parameters constrained
Δρ_max_, Δρ_min_ (e Å^−3^)	0.49, −0.38

Computer programs: *CrystalClear* (Rigaku/MSC, 2008 ▸), *SHELXS97* (Sheldrick, 2008 ▸), *SHELXL2014* (Sheldrick, 2015 ▸) and *Mercury* (Macrae *et al.*, 2006 ▸).

## supplementary crystallographic information

### Crystal data

**Table:**

C_24_H_25_NO_9_	*D*_x_ = 1.367 Mg m^−^^3^
*M**_r_* = 471.46	Melting point = 366–369 K
Monoclinic, *P*2_1_/*n*	Mo *K*α radiation, λ = 0.71073 Å
*a* = 11.0111 (7) Å	Cell parameters from 39929 reflections
*b* = 13.1762 (8) Å	θ = 3.0–31.5°
*c* = 15.8196 (9) Å	µ = 0.11 mm^−^^1^
β = 93.802 (2)°	*T* = 103 K
*V* = 2290.1 (2) Å^3^	Block, colorless
*Z* = 4	0.45 × 0.38 × 0.08 mm
*F*(000) = 992	

### Data collection

**Table:**

R-AXIS-RAPID diffractometer	7609 independent reflections
Radiation source: Sealed Tube	6054 reflections with *I* > 2σ(*I*)
Graphite monochromator	*R*_int_ = 0.046
Detector resolution: 10.0000 pixels mm^-1^	θ_max_ = 31.5°, θ_min_ = 3.0°
dtprofit.ref scans	*h* = −16→16
Absorption correction: empirical (using intensity measurements) Higashi (2002). Numerical Absorption Correction: *NUMABS*	*k* = −19→19
*T*_min_ = 0.957, *T*_max_ = 0.979	*l* = −23→23
67635 measured reflections	

### Refinement

**Table:**

Refinement on *F*^2^	Primary atom site location: difference Fourier map
Least-squares matrix: full	Secondary atom site location: difference Fourier map
*R*[*F*^2^ > 2σ(*F*^2^)] = 0.049	Hydrogen site location: difference Fourier map
*wR*(*F*^2^) = 0.128	H-atom parameters constrained
*S* = 1.12	*w* = 1/[σ^2^(*F*_o_^2^) + (0.0518*P*)^2^ + 0.9253*P*] where *P* = (*F*_o_^2^ + 2*F*_c_^2^)/3
7609 reflections	(Δ/σ)_max_ = 0.001
310 parameters	Δρ_max_ = 0.49 e Å^−^^3^
0 restraints	Δρ_min_ = −0.38 e Å^−^^3^

### Special details

**Table:**

Geometry. All e.s.d.'s (except the e.s.d. in the dihedral angle between two l.s. planes) are estimated using the full covariance matrix. The cell e.s.d.'s are taken into account individually in the estimation of e.s.d.'s in distances, angles and torsion angles; correlations between e.s.d.'s in cell parameters are only used when they are defined by crystal symmetry. An approximate (isotropic) treatment of cell e.s.d.'s is used for estimating e.s.d.'s involving l.s. planes.

### Fractional atomic coordinates and isotropic or equivalent isotropic displacement parameters (Å^2^)

**Table:**

	*x*	*y*	*z*	*U*_iso_*/*U*_eq_	
O3	0.16456 (8)	0.30912 (6)	0.25349 (6)	0.01897 (17)	
O5	0.26884 (8)	0.41900 (7)	0.14208 (6)	0.02136 (18)	
O6	0.21898 (8)	0.56611 (7)	0.20333 (6)	0.02246 (18)	
O2	0.14316 (8)	0.54562 (6)	0.36420 (6)	0.02048 (18)	
O4	0.01727 (9)	0.37984 (8)	0.16696 (6)	0.0278 (2)	
O1	0.01500 (8)	0.41220 (7)	0.35300 (7)	0.0267 (2)	
C11	0.67021 (11)	0.06374 (9)	0.29676 (8)	0.0205 (2)	
H11	0.6453	−0.0032	0.2816	0.025*	
C5	0.40905 (10)	0.31827 (9)	0.29982 (8)	0.0177 (2)	
H5B	0.3767	0.2484	0.3036	0.021*	
H5A	0.4138	0.3351	0.2391	0.021*	
C8	0.74760 (11)	0.25968 (9)	0.34224 (8)	0.0211 (2)	
H8	0.7733	0.3264	0.3576	0.025*	
O7	0.56808 (9)	0.39344 (8)	0.38850 (7)	0.0299 (2)	
C4	0.32199 (10)	0.39265 (9)	0.33974 (7)	0.0165 (2)	
H4	0.3639	0.4599	0.3448	0.020*	
C10	0.79207 (11)	0.08750 (9)	0.31234 (8)	0.0194 (2)	
N1	0.88210 (10)	0.00521 (8)	0.30793 (7)	0.0236 (2)	
O8	0.98968 (9)	0.02854 (9)	0.30729 (9)	0.0407 (3)	
C1	0.10634 (10)	0.45486 (9)	0.33559 (8)	0.0186 (2)	
C2	0.20283 (10)	0.40756 (8)	0.28145 (7)	0.0167 (2)	
O9	0.84527 (10)	−0.08248 (8)	0.30556 (8)	0.0339 (2)	
C16	0.23200 (12)	0.30653 (10)	0.59164 (8)	0.0239 (2)	
H16	0.2111	0.2884	0.6470	0.029*	
C6	0.53495 (10)	0.32257 (9)	0.34362 (8)	0.0192 (2)	
C3	0.22914 (10)	0.47531 (9)	0.20474 (8)	0.0179 (2)	
C7	0.62319 (10)	0.23832 (9)	0.32841 (7)	0.0180 (2)	
C13	0.29301 (10)	0.36000 (9)	0.42848 (7)	0.0180 (2)	
C23	0.07300 (11)	0.30567 (10)	0.19098 (8)	0.0220 (2)	
C18	0.24448 (12)	0.26431 (9)	0.44386 (8)	0.0231 (2)	
H18	0.2321	0.2170	0.3988	0.028*	
C14	0.31184 (11)	0.42777 (9)	0.49593 (8)	0.0203 (2)	
H14	0.3463	0.4925	0.4865	0.024*	
C20	0.11540 (16)	0.69755 (11)	0.44259 (10)	0.0331 (3)	
H20C	0.0675	0.7306	0.4848	0.040*	
H20A	0.1071	0.7358	0.3894	0.040*	
H20B	0.2012	0.6956	0.4633	0.040*	
C9	0.83308 (11)	0.18426 (10)	0.33364 (8)	0.0217 (2)	
H9	0.9176	0.1983	0.3421	0.026*	
C12	0.58535 (11)	0.14101 (9)	0.30400 (8)	0.0200 (2)	
H12	0.5012	0.1274	0.2922	0.024*	
C24	0.05811 (13)	0.20038 (11)	0.15718 (10)	0.0299 (3)	
H24A	0.1384	0.1711	0.1493	0.036*	
H24C	0.0103	0.2021	0.1027	0.036*	
H24B	0.0159	0.1588	0.1974	0.036*	
C19	0.06994 (12)	0.59136 (10)	0.42759 (9)	0.0252 (3)	
H19A	0.0781	0.5519	0.4809	0.030*	
H19B	−0.0169	0.5923	0.4070	0.030*	
C17	0.21419 (13)	0.23829 (10)	0.52514 (9)	0.0257 (3)	
H17	0.1810	0.1732	0.5352	0.031*	
C15	0.28063 (12)	0.40142 (10)	0.57657 (8)	0.0229 (2)	
H15	0.2926	0.4486	0.6217	0.027*	
C21	0.28528 (14)	0.47311 (10)	0.06304 (8)	0.0270 (3)	
H21A	0.3402	0.4339	0.0282	0.032*	
H21B	0.3233	0.5400	0.0757	0.032*	
C22	0.16416 (16)	0.48792 (13)	0.01442 (10)	0.0390 (4)	
H22B	0.1765	0.5225	−0.0392	0.047*	
H22C	0.1112	0.5292	0.0480	0.047*	
H22A	0.1261	0.4217	0.0028	0.047*	

### Atomic displacement parameters (Å^2^)

**Table:**

	*U*^11^	*U*^22^	*U*^33^	*U*^12^	*U*^13^	*U*^23^
O3	0.0175 (4)	0.0147 (4)	0.0243 (4)	−0.0015 (3)	−0.0016 (3)	−0.0012 (3)
O5	0.0267 (4)	0.0191 (4)	0.0185 (4)	0.0018 (3)	0.0032 (3)	−0.0001 (3)
O6	0.0272 (4)	0.0168 (4)	0.0233 (4)	0.0016 (3)	0.0016 (3)	0.0011 (3)
O2	0.0209 (4)	0.0170 (4)	0.0241 (4)	0.0014 (3)	0.0057 (3)	−0.0016 (3)
O4	0.0223 (4)	0.0271 (5)	0.0331 (5)	0.0028 (4)	−0.0049 (4)	−0.0010 (4)
O1	0.0201 (4)	0.0263 (5)	0.0343 (5)	−0.0025 (3)	0.0062 (4)	−0.0022 (4)
C11	0.0201 (5)	0.0181 (5)	0.0232 (6)	−0.0002 (4)	0.0013 (4)	−0.0003 (4)
C5	0.0156 (5)	0.0168 (5)	0.0205 (5)	0.0010 (4)	−0.0003 (4)	−0.0020 (4)
C8	0.0174 (5)	0.0205 (5)	0.0249 (6)	−0.0002 (4)	−0.0010 (4)	−0.0023 (5)
O7	0.0210 (4)	0.0279 (5)	0.0401 (6)	0.0008 (4)	−0.0030 (4)	−0.0160 (4)
C4	0.0153 (5)	0.0161 (5)	0.0181 (5)	0.0005 (4)	0.0003 (4)	−0.0011 (4)
C10	0.0189 (5)	0.0207 (5)	0.0186 (5)	0.0036 (4)	0.0019 (4)	0.0006 (4)
N1	0.0219 (5)	0.0233 (5)	0.0257 (5)	0.0054 (4)	0.0012 (4)	0.0010 (4)
O8	0.0191 (5)	0.0328 (6)	0.0699 (8)	0.0054 (4)	−0.0001 (5)	−0.0033 (5)
C1	0.0160 (5)	0.0170 (5)	0.0226 (5)	0.0022 (4)	−0.0007 (4)	0.0014 (4)
C2	0.0165 (5)	0.0131 (5)	0.0203 (5)	−0.0006 (4)	0.0004 (4)	−0.0002 (4)
O9	0.0326 (5)	0.0206 (4)	0.0494 (6)	0.0044 (4)	0.0099 (5)	0.0007 (4)
C16	0.0260 (6)	0.0273 (6)	0.0188 (5)	0.0031 (5)	0.0034 (4)	0.0019 (5)
C6	0.0161 (5)	0.0196 (5)	0.0217 (5)	0.0000 (4)	0.0005 (4)	−0.0022 (4)
C3	0.0154 (5)	0.0180 (5)	0.0201 (5)	0.0002 (4)	−0.0011 (4)	0.0003 (4)
C7	0.0167 (5)	0.0186 (5)	0.0185 (5)	0.0002 (4)	−0.0005 (4)	−0.0007 (4)
C13	0.0179 (5)	0.0172 (5)	0.0187 (5)	0.0020 (4)	−0.0001 (4)	−0.0002 (4)
C23	0.0177 (5)	0.0233 (6)	0.0250 (6)	−0.0025 (4)	0.0003 (4)	−0.0023 (5)
C18	0.0285 (6)	0.0183 (5)	0.0227 (6)	−0.0003 (4)	0.0034 (5)	−0.0017 (5)
C14	0.0194 (5)	0.0196 (5)	0.0216 (5)	0.0010 (4)	−0.0015 (4)	−0.0007 (4)
C20	0.0461 (9)	0.0238 (6)	0.0304 (7)	−0.0004 (6)	0.0110 (6)	−0.0066 (5)
C9	0.0156 (5)	0.0240 (6)	0.0252 (6)	0.0004 (4)	−0.0017 (4)	−0.0013 (5)
C12	0.0163 (5)	0.0204 (5)	0.0232 (6)	−0.0006 (4)	0.0003 (4)	−0.0007 (4)
C24	0.0253 (6)	0.0257 (6)	0.0382 (8)	−0.0053 (5)	−0.0023 (5)	−0.0080 (6)
C19	0.0257 (6)	0.0235 (6)	0.0273 (6)	0.0041 (5)	0.0088 (5)	−0.0021 (5)
C17	0.0318 (7)	0.0204 (6)	0.0255 (6)	−0.0013 (5)	0.0060 (5)	0.0024 (5)
C15	0.0234 (6)	0.0252 (6)	0.0198 (5)	0.0020 (5)	−0.0008 (4)	−0.0036 (5)
C21	0.0379 (7)	0.0244 (6)	0.0191 (6)	0.0015 (5)	0.0058 (5)	0.0016 (5)
C22	0.0491 (9)	0.0432 (9)	0.0232 (7)	−0.0088 (7)	−0.0089 (6)	0.0033 (6)

### Geometric parameters (Å, º)

**Table:**

O1—C1	1.2000 (15)	C16—C17	1.3879 (19)
O2—C1	1.3323 (14)	C17—C18	1.3925 (19)
O2—C19	1.4581 (16)	C19—C20	1.500 (2)
O3—C2	1.4251 (13)	C21—C22	1.507 (2)
O3—C23	1.3653 (15)	C23—C24	1.492 (2)
O4—C23	1.2021 (17)	C4—H4	1.0000
O5—C3	1.3348 (15)	C5—H5A	0.9900
O5—C21	1.4610 (16)	C5—H5B	0.9900
O6—C3	1.2017 (15)	C8—H8	0.9500
O7—C6	1.2144 (16)	C9—H9	0.9500
O8—N1	1.2245 (15)	C11—H11	0.9500
O9—N1	1.2243 (15)	C12—H12	0.9500
N1—C10	1.4739 (16)	C14—H14	0.9500
C1—C2	1.5400 (16)	C15—H15	0.9500
C2—C3	1.5492 (16)	C16—H16	0.9500
C2—C4	1.5653 (16)	C17—H17	0.9500
C4—C5	1.5358 (16)	C18—H18	0.9500
C4—C13	1.5222 (16)	C19—H19A	0.9900
C5—C6	1.5092 (16)	C19—H19B	0.9900
C6—C7	1.5051 (16)	C20—H20A	0.9800
C7—C8	1.4014 (16)	C20—H20B	0.9800
C7—C12	1.3948 (17)	C20—H20C	0.9800
C8—C9	1.3816 (17)	C21—H21A	0.9900
C9—C10	1.3866 (18)	C21—H21B	0.9900
C10—C11	1.3840 (17)	C22—H22A	0.9800
C11—C12	1.3918 (17)	C22—H22B	0.9800
C13—C14	1.3963 (17)	C22—H22C	0.9800
C13—C18	1.3970 (17)	C24—H24A	0.9800
C14—C15	1.3872 (18)	C24—H24B	0.9800
C15—C16	1.3870 (19)	C24—H24C	0.9800
			
C1—O2—C19	115.78 (9)	C4—C5—H5A	109.00
C2—O3—C23	116.36 (9)	C4—C5—H5B	109.00
C3—O5—C21	115.44 (10)	C6—C5—H5A	109.00
O8—N1—O9	123.73 (12)	C6—C5—H5B	109.00
O8—N1—C10	118.02 (11)	H5A—C5—H5B	108.00
O9—N1—C10	118.25 (11)	C7—C8—H8	120.00
O1—C1—O2	125.68 (11)	C9—C8—H8	120.00
O1—C1—C2	123.90 (11)	C8—C9—H9	121.00
O2—C1—C2	110.28 (9)	C10—C9—H9	121.00
O3—C2—C1	109.89 (9)	C10—C11—H11	121.00
O3—C2—C3	110.37 (9)	C12—C11—H11	121.00
O3—C2—C4	106.72 (9)	C7—C12—H12	120.00
C1—C2—C3	111.98 (9)	C11—C12—H12	120.00
C1—C2—C4	107.79 (9)	C13—C14—H14	120.00
C3—C2—C4	109.93 (9)	C15—C14—H14	120.00
O5—C3—O6	125.05 (12)	C14—C15—H15	120.00
O5—C3—C2	110.42 (10)	C16—C15—H15	120.00
O6—C3—C2	124.49 (11)	C15—C16—H16	120.00
C2—C4—C5	111.07 (9)	C17—C16—H16	120.00
C2—C4—C13	111.10 (9)	C16—C17—H17	120.00
C5—C4—C13	111.94 (10)	C18—C17—H17	120.00
C4—C5—C6	111.51 (10)	C13—C18—H18	120.00
O7—C6—C5	121.90 (11)	C17—C18—H18	120.00
O7—C6—C7	119.24 (10)	O2—C19—H19A	110.00
C5—C6—C7	118.82 (10)	O2—C19—H19B	110.00
C6—C7—C8	117.49 (10)	C20—C19—H19A	110.00
C6—C7—C12	122.56 (10)	C20—C19—H19B	110.00
C8—C7—C12	119.92 (11)	H19A—C19—H19B	109.00
C7—C8—C9	120.36 (11)	C19—C20—H20A	109.00
C8—C9—C10	118.18 (11)	C19—C20—H20B	109.00
N1—C10—C9	118.60 (11)	C19—C20—H20C	109.00
N1—C10—C11	118.19 (10)	H20A—C20—H20B	109.00
C9—C10—C11	123.20 (11)	H20A—C20—H20C	109.00
C10—C11—C12	117.92 (11)	H20B—C20—H20C	110.00
C7—C12—C11	120.36 (11)	O5—C21—H21A	110.00
C4—C13—C14	119.63 (10)	O5—C21—H21B	110.00
C4—C13—C18	121.45 (10)	C22—C21—H21A	110.00
C14—C13—C18	118.90 (11)	C22—C21—H21B	110.00
C13—C14—C15	120.63 (11)	H21A—C21—H21B	108.00
C14—C15—C16	120.38 (12)	C21—C22—H22A	109.00
C15—C16—C17	119.37 (12)	C21—C22—H22B	109.00
C16—C17—C18	120.66 (12)	C21—C22—H22C	109.00
C13—C18—C17	120.06 (11)	H22A—C22—H22B	109.00
O2—C19—C20	107.49 (11)	H22A—C22—H22C	109.00
O5—C21—C22	110.13 (12)	H22B—C22—H22C	109.00
O3—C23—O4	122.60 (12)	C23—C24—H24A	109.00
O3—C23—C24	110.42 (11)	C23—C24—H24B	109.00
O4—C23—C24	126.94 (12)	C23—C24—H24C	109.00
C2—C4—H4	107.00	H24A—C24—H24B	109.00
C5—C4—H4	108.00	H24A—C24—H24C	109.00
C13—C4—H4	107.00	H24B—C24—H24C	109.00
			
C19—O2—C1—O1	5.55 (18)	C1—C2—C3—O5	−153.75 (9)
C19—O2—C1—C2	−170.22 (10)	C2—C4—C13—C14	−109.31 (12)
C1—O2—C19—C20	−172.46 (11)	C5—C4—C13—C18	−55.97 (14)
C23—O3—C2—C3	−50.41 (13)	C13—C4—C5—C6	−69.13 (12)
C23—O3—C2—C1	73.57 (12)	C2—C4—C5—C6	166.04 (9)
C23—O3—C2—C4	−169.83 (9)	C5—C4—C13—C14	125.88 (11)
C2—O3—C23—C24	169.59 (10)	C2—C4—C13—C18	68.84 (14)
C2—O3—C23—O4	−8.34 (17)	C4—C5—C6—O7	−17.48 (17)
C3—O5—C21—C22	−78.91 (13)	C4—C5—C6—C7	164.86 (10)
C21—O5—C3—C2	174.18 (10)	C5—C6—C7—C8	156.98 (11)
C21—O5—C3—O6	−8.16 (17)	O7—C6—C7—C12	157.30 (12)
O8—N1—C10—C9	−13.61 (18)	C5—C6—C7—C12	−24.98 (17)
O9—N1—C10—C11	−12.60 (18)	O7—C6—C7—C8	−20.74 (17)
O9—N1—C10—C9	166.02 (12)	C8—C7—C12—C11	2.74 (18)
O8—N1—C10—C11	167.78 (13)	C12—C7—C8—C9	−1.43 (18)
O2—C1—C2—C4	62.12 (12)	C6—C7—C8—C9	176.67 (11)
O1—C1—C2—C4	−113.74 (13)	C6—C7—C12—C11	−175.26 (11)
O2—C1—C2—O3	178.06 (9)	C7—C8—C9—C10	−0.93 (18)
O1—C1—C2—C3	125.24 (13)	C8—C9—C10—C11	2.08 (19)
O2—C1—C2—C3	−58.90 (13)	C8—C9—C10—N1	−176.45 (11)
O1—C1—C2—O3	2.19 (16)	C9—C10—C11—C12	−0.80 (19)
O3—C2—C3—O5	−30.98 (12)	N1—C10—C11—C12	177.74 (11)
C1—C2—C4—C5	162.98 (9)	C10—C11—C12—C7	−1.63 (18)
C1—C2—C4—C13	37.68 (12)	C4—C13—C14—C15	176.90 (11)
O3—C2—C3—O6	151.34 (11)	C18—C13—C14—C15	−1.30 (18)
C4—C2—C3—O6	−91.21 (13)	C4—C13—C18—C17	−177.30 (11)
C3—C2—C4—C13	159.98 (9)	C14—C13—C18—C17	0.86 (18)
O3—C2—C4—C13	−80.32 (11)	C13—C14—C15—C16	1.06 (19)
C3—C2—C4—C5	−74.72 (11)	C14—C15—C16—C17	−0.4 (2)
C4—C2—C3—O5	86.48 (11)	C15—C16—C17—C18	−0.1 (2)
O3—C2—C4—C5	44.98 (12)	C16—C17—C18—C13	−0.2 (2)
C1—C2—C3—O6	28.57 (16)		

### Hydrogen-bond geometry (Å, º)

*Cg* is the centroid of the C7–C12 ring.

**Table:**

*D*—H···*A*	*D*—H	H···*A*	*D*···*A*	*D*—H···*A*
C5—H5*A*···O5	0.99	2.41	3.1403 (15)	130
C11—H11···O4^i^	0.95	2.54	3.2588 (16)	133
C12—H12···O6^i^	0.95	2.56	3.4879 (15)	165
C15—H15···O7^ii^	0.95	2.60	3.2038 (17)	122
C24—H24*B*···O8^iii^	0.98	2.47	3.402 (2)	158
C16—H16···*Cg*^iv^	0.95	2.81	3.6550 (14)	149

Symmetry codes: (i) −*x*+1/2, *y*−1/2, −*z*+1/2; (ii) −*x*+1, −*y*+1, −*z*+1; (iii) *x*−1, *y*, *z*; (iv) *x*−1/2, −*y*+1/2, *z*+1/2.
